# Societal implications of a changing Arctic Ocean

**DOI:** 10.1007/s13280-021-01601-2

**Published:** 2021-07-19

**Authors:** Henry P. Huntington, Andrey Zagorsky, Bjørn P. Kaltenborn, Hyoung Chul Shin, Jackie Dawson, Maija Lukin, Parnuna Egede Dahl, Peiqing Guo, David N. Thomas

**Affiliations:** 1 Alaska, USA; 2 Moscow, Russia; 3 NINA, Lillehammer, Norway; 4 Incheon, Republic of Korea; 5 Ottawa, Canada; 6 Kotzebue, USA; 7 Koege, Denmark; 8 Qingdao, China; 9 University of Helsinki, P.O. Box 65 (Viikinkaari 1), 00014 Helsinki, Finland

**Keywords:** Arctic Ocean, Climate change, Ecology, Geopolitics, Indigenous Peoples, Sea ice

## Abstract

The Arctic Ocean is undergoing rapid change: sea ice is being lost, waters are warming, coastlines are eroding, species are moving into new areas, and more. This paper explores the many ways that a changing Arctic Ocean affects societies in the Arctic and around the world. In the Arctic, Indigenous Peoples are again seeing their food security threatened and cultural continuity in danger of disruption. Resource development is increasing as is interest in tourism and possibilities for trans-Arctic maritime trade, creating new opportunities and also new stresses. Beyond the Arctic, changes in sea ice affect mid-latitude weather, and Arctic economic opportunities may re-shape commodities and transportation markets. Rising interest in the Arctic is also raising geopolitical tensions about the region. What happens next depends in large part on the choices made within and beyond the Arctic concerning global climate change and industrial policies and Arctic ecosystems and cultures.

## Introduction

Rapid environmental changes in the Arctic Ocean have brought a considerable attention to the region in recent years (e.g., Huntington et al. 2020; Wassmann et al. 2020). Most notable is the loss of sea ice, a highly visible indicator of global climate change (Meredith and Sommerkorn 2019; Slater et al. 2021) and an initiator of further warming as open water absorbs sunlight that had previously been reflected from snow and ice (Perovich 2017). In addition, waters are warming (Thoman et al. 2020), species are moving into new areas (Moore et al. 2014), the structure of the food web is changing (Gall et al. 2017), coastlines are eroding (Marino 2015), and more.

The Changing Arctic Ocean Programme (2017 to 2021; https://www.changing-arctic-ocean.ac.uk/) was a major international study of the implications of the region’s changing marine biology and biogeochemistry. Specifically, it aimed to help understand how change in the physical environment (ice and ocean) will affect large-scale ecosystem structure and functioning, examine potential major impacts, and provide projections for future ecosystem services. It comprised 16 research projects funded by the United Kingdom’s Natural Environment Research Council and the German Federal Ministry of Education and Research. Our paper is intended to present the societal context for the results of the Changing Arctic Ocean Programme, some of which are reported in other papers in this special issue (Thomas et al. 2022).

Today’s interest in the Arctic Ocean may be high, but it is far from unprecedented. The Arctic has long fascinated European societies, from myths about a realm of warmth and plenty beyond the ice (Simmonds 1852) to the climax of the novel *Frankenstein* (Shelley 1818), to the geographical and commercial lure of the Northwest and Northeast Passages (Nordenskiöld 1880; Berton 1988). Over 250 years ago, Mikhail Lomonosov (1952) recognized the geopolitical significance of the Arctic for its resources and its transportation routes. Recent environmental changes have boosted, but not created, dreams of Arctic potential and concerns for Arctic and global well-being.

For far longer, the Arctic Ocean has been the home of Indigenous Peoples (Fitzhugh and Crowell 1988), with their own myths and legends (Rasmussen 1921) as well as practical and detailed knowledge of the environment (Petersen 2010) and a desire to make their own decisions about how to live their lives and organize their societies (Watt-Cloutier 2016). Their interests and rights have often clashed with the ambitions of newcomers, especially through the many aspects of forcible colonization that continue to affect the Arctic today (Stuhl 2016). Environmental change and its implications must be understood in this longer societal setting (Mustonen and Van Dam 2021).

Our look at the societal implications of a changing Arctic Ocean first considers the well-being of those who live in the Arctic. Second, we look at effects on those who live elsewhere in the world. Third, we examine implications for global affairs, before concluding with a discussion of where we are now and the choices that will determine where we are headed.

## Implications for well-being in the Arctic

Some 4 million people live in the Arctic, including the many distinct Indigenous Peoples who make up about 10% of the Arctic population (Larsen and Fondahl 2015). Coastal communities are found along the shores of the Arctic Ocean and its adjacent seas. Livelihoods include commercial fishing, transportation, mineral and hydrocarbon extraction, and traditional practices of hunting, fishing, herding, and gathering (Glomsrød et al. 2017). The ocean is also the primary route for delivering supplies such as fuel, non-perishable foods, building materials, and other heavy items to many communities (AMSA 2009). Changes in the ocean can affect all these activities.

A major concern for Arctic residents is food security (CCA 2014), defined as having reliable access to nutritious foods of one’s choosing. For Arctic Indigenous Peoples, food security includes food sovereignty, the ability to control one’s own access to food (ICC 2015), for example, by continuing traditional practices and recognizing the connections among sea ice, food, and identity (Gearheard et al. 2013). A changing environment does not necessarily mean a loss of food security, as new species may become available or new hunting seasons become possible (Huntington et al. 2017), but in practice, the changes to date have tended to undermine food security by reducing hunting and fishing opportunities as well as interfering with cultural continuity (CCA 2014).

Loss of sea ice has increased hazards associated with marine mammal hunting, to the point that some families no longer teach their children to travel on the ice in winter (Iñupiaq co-author Maija Lukin, personal experience). Sea ice loss has also facilitated increased access for commercial and non-commercial shipping, which has cascading implications for food security resulting from ship-source underwater noise and ship strikes impacting important marine mammal species such as narwhal (*Monodon monoceros*), bowhead (*Balaena mysticetus*), and beluga whales (*Delphinapterus leucas*) (Halliday et al. 2018; Huntington et al. 2021). The effects of these changes are often exacerbated by regulatory constraints such as limited hunting seasons or prohibiting the use of certain species. These and other measures perpetuate colonial legacies, limit self-determination, constrain flexibility, and challenge local capacity for sustainability (Huntington et al. 2017).

The loss of sea ice is often heralded as permitting greater access to the Arctic, for fisheries, resource extraction, transportation, and the potential for trans-Arctic trade (e.g., Pizzolato et al. 2016; Bennett et al. 2020). Over the past decade, there has been a 79% increase in total transit tonnage along the Northern Sea Route (Babin et al. 2020), and a tripling of ship traffic by kilometers traveled along the Northwest Passage (Dawson et al. 2018). These trends are expected to continue as sea ice decreases further, and even the Transpolar Route directly across the Arctic Basin may become a viable option by mid-century (Melia et al. 2016).

Changing water temperatures are also affecting the distribution of Atlantic cod (*Gadus morhua*), Atlantic halibut (*Hippoglossus hippoglossus*), walleye pollock (*Gadus chalcogrammus*), and other fish species (Hollowed et al. 2013; Fossheim et al. 2015), altering coastal opportunities for better or for worse (Hamilton et al. 2003). While a strong economy can be good for community well-being and can be harnessed to support the continuation of cultural activities (Baffrey and Huntington 2010), industrial activity such as commercial shipping and offshore oil and gas extraction can disrupt Arctic marine ecosystems and cultural practices (AMSA 2009; AMAP 2018). Navigating between the perils of poverty and the hazards of environmental degradation remains a major challenge for Arctic regions.

Tourism is an example of the connections between the Arctic marine environment and global society. Svalbard, for example, has attracted tourists since the 1800s (Arlov 2003), due largely to relative ease of access. Prior to the COVID-19 pandemic, the tourism sector provided the equivalent of 618 full-time jobs out of a total of 1518 in Longyearbyen, Svalbard, a major shift from earlier emphasis on coal mining (Hovelsrud et al. 2020; Statistics Norway 2020). Climate change is creating less predictable weather and, thus, greater risks for residents and visitors. Less sea ice means more polar bears on land, leading to more bear-human encounters (Wilder et al. 2017). Similar questions arise elsewhere as interest in Arctic tourism increases, leading to policy debates about sustainability and cultural and environmental impacts (e.g., Kaltenborn et al. 2020; Olsen et al. 2020).

In addition to changes in the sea, many coastal communities in the Arctic, especially in western North America, are built on low-lying coastlines made of permafrost (Melvin et al. 2017). Rising temperatures thaw the permafrost, and lack of sea ice increases exposure to storm waves, leading to rapid coastal erosion that threatens many communities, entailing high costs and possibly relocation (GAO 2003; Marino 2015). Sea level rise will only hasten these processes, which may lead to extensive dislocation and cultural loss, for example, through the exposure and destruction of archeological sites (Hollesen et al. 2018).

The importance of environmental conservation in the Arctic has grown while also becoming more complex in relation to economic aspirations, human rights, and Indigenous self-determination (Mustonen and Ford 2013). Some protective measures have been developed, for example, marine protected areas in the western Canadian Arctic (Fisheries and Oceans Canada 2016, 2019) in cooperation with Inuvialuit communities and shipping routes and areas to be avoided in the Bering Strait (IMO 2018). The 2018 “Agreement to prevent unregulated high seas fisheries in the central Arctic Ocean” (CAO Fisheries Agreement) is a landmark in precautionary international action, but at the same time, some countries are eyeing the prospects for expanding their fisheries northwards (Rosen 2020). The North Water Polynya, located in Baffin Bay between Canada and Greenland, is one of the largest biological hotspots in the Arctic but is projected to become weaker and less stable (AMAP 2018), affecting the ecosystem and Inuit communities on both sides of the bay. Accordingly, Inuit is leading efforts to establish an Indigenous Protected Area for the North Water Polynya to protect it from industrial activities while allowing Indigenous subsistence hunting practices to continue (Pikialasorsuaq Commission 2017).

As far reaching as the effects of a changing Arctic Ocean may be on Arctic societies and cultures, they are not the only challenges facing Arctic residents (Huntington et al. 2019). Cultural change, limited access to health care, economic assimilation and marginalization, and other factors vary greatly around the Arctic and create urgent needs that can outweigh long-term considerations (Huntington and Eerkes-Medrano 2017). Understanding a changing Arctic Ocean is important to understanding what Arctic communities and peoples face but not sufficient without attention to the rest of the social-ecological systems and megatrends (Nordic Council of Ministers 2011) of which they are part.

## Implications for well-being beyond the Arctic

A changing Arctic Ocean has effects far beyond the waters in question. The Arctic is part of the global ocean and climate systems and is increasingly connected to global society as well. Many who had never given the Arctic a second thought are now affected in ways large and small by what is taking place in the North. Over 200 years ago, the British naval captain Edward Parry (quoted in O’Brian 1997) noted that a change in sea ice near Greenland affected the quality of whale oil and led to crop failures and colder summers far to the south. Similar connections can be seen today.

Mid-latitude weather in the Northern Hemisphere has been widely affected as Arctic sea ice loss has changed the patterns of the jet stream (Francis and Vavrus 2015; Ge et al. 2020; Zou et al. 2020). This effect has produced cold winters in North America and East Asia (Kug et al. 2015). Such teleconnections (Mao et al. 2011; Zhao et al. 2019) are among the reasons that China considers itself a “near Arctic state.” Changes in the movements of water masses in the North Atlantic, driven in part by warmer Arctic waters, may affect global ocean circulation including the Gulf Stream that keeps Europe warmer than its latitude would suggest (Caesar et al. 2021). An understanding of such meteorological connections is essential for accurate weather prediction and for long-term planning and preparation for extreme weather events.

Climatologically, loss of Arctic sea ice amplifies global warming by allowing more solar energy to be retained instead of reflected (McGuire et al. 2006). Thus, the effects of sea ice loss amplify the effects of increased atmospheric carbon dioxide levels (Bony et al. 2006). Economic estimates of the climate cost of sea ice loss run into the tens and hundreds of billions of dollars (Euskirchen et al. 2013). The change may be occurring in Arctic waters, but the entire globe will feel—and pay for—the effects.

Changing access to Arctic resources may also affect global commodities markets and create economic benefits. Liquefied natural gas from northern Siberia is already being shipped to East Asia and Europe, providing energy while tapping into the shipbuilding prowess of the Republic of Korea (The Maritime Executive 2016) and helping Russia maintain the Northern Sea Route as a viable transportation corridor (Putin 2018). Shipping through the Arctic, as distinct from shipping to and from Arctic destinations, has also attracted great interest, not yet a huge increase in vessel traffic (Staalesen 2020), due in part to variable ice conditions that reduce predictability and retain hazards (Pizzolato et al. 2016). For the longer term, Arctic shipping features prominently in strategies such as China’s One Belt One Road initiative (Sum 2019), though no actual Arctic projects have been undertaken thus far.

## Implications for global affairs

A changing Arctic may also shift international relations by raising the stakes associated with access to and control of Arctic waters. Apart from relatively minor maritime boundary disputes, international boundaries in the Arctic are fixed and universally recognized (Spohr and Hamilton 2020; Zagorskij 2020). The main unsettled issues concern the extended continental shelf and the overlapping claims of several countries to seabed resources (Antsygina et al. 2020; Zagorskij 2020). In a context of uncertainty, many countries are pursuing influence and engagement in a variety of ways, which include military, economic, scientific, and other forms. A changing Arctic Ocean is only likely to exacerbate both the uncertainty and the range and magnitude of responses around the Arctic and the world.

The Arctic has at times seen international conflict and its trappings before, as causes and results of change. The last shots of the American Civil War were fired off the northwestern coast of Alaska in the 1860s (Bockstoce 1986). In the 1950s, the United States built an extensive radar detection system from Greenland to the Bering Strait (Chasen 1967), just as the Soviet Union used Novaya Zemlya to test nuclear weapons (Strand et al. 1998). Today, Russia maintains major naval bases on the Kola Peninsula, north of the Arctic Circle (Zagorskiy 2019), and its Northern Fleet was raised to equal status as a fifth major military district in the country as of the start of 2021 (Putin 2020). Militarization remains a concern for many, for example, in the United States’ claimed justification for increasing its military presence in the warmer parts of the region as a response to other countries’ actions (Office for the Undersecretary of Defense Policy 2019). Moreover, nuclear submarines from the major powers can operate in these waters, with the noise from moving ice helping prevent sonar detection of submarines (Palma et al. 2019), providing a further incentive to use the Arctic Ocean for military purposes.

Arctic resources and access are behind at least some of these concerns (Kaltenborn et al. 2020; Spohr and Hamilton 2020), as can be seen in two recent and prominent examples. The Polar Silk Road, the northern component of China’s vision for One Belt One Road, includes a spate of infrastructure proposals across northern Eurasia (Sum 2019) and entangles economic opportunity through shorter shipping routes with geopolitical aspirations (Su and Huntington 2021). The difficulty of distinguishing between the interests of Chinese companies and those of the central government will make it all the harder to tell which is the overriding factor (Klinger and Muldavin 2019), at least for those outside of China. The European Union and three Asian states joined six Arctic states to complete the CAO Fisheries Agreement, a recognition of major non-Arctic interests in Arctic marine resources (Vylegzhanin et al. 2020). Should fish species shift farther north and increase in abundance, pressure will likewise increase to turn a fishing moratorium into a fisheries management regime. Political disputes over other matters may clash with a need for economic and other partnerships in the Arctic development.

Arctic scientific research is relevant locally and regionally and is also growing in significance as a mode of international relations (Royal Society 2010), in response to change and as a way of exerting influence. Significantly, science is prominently featured in the Arctic policy statements of many non-Arctic countries such as China, India, Japan, and Korea (Headquarters for Ocean Policy 2015; State Council Information Office 2018; Ministry of Oceans and Fisheries 2019; Rej 2021), as is also the case for Arctic states. Arctic research is costly, but the benefits can outweigh the cost (Dobricic et al. 2018). The Arctic Council’s Agreement on Enhancing International Arctic Scientific Cooperation is an important step in this regard, as is the commitment to the joint scientific program being set up under the CAO Fisheries Agreement. Even high-quality science, however, will not necessarily translate to effective policies and practices without broader efforts to engage society and its leaders in recognizing and addressing both threats and opportunities posed by change. This special collection of research results from a major international program is a case in point, highlighting important findings, and still needing to connect those findings with the needs of global society (Thomas et al. 2022).

International relations are of course shaped by circumstances around the world. The Arctic has in many ways remained apart from other clashes. In 1988, the Soviet Union sent icebreakers to assist Alaskans trying to free gray whales trapped in ice near Point Barrow (Russell 2004), even as the Cold War continued. In 2018, Russia and the United States cooperated to propose Bering Strait shipping routes to the International Maritime Organization (IMO 2018), despite clashing over Crimea and other issues. The Arctic states have shown an exemplary will to cooperate through the work of the Arctic Council while keeping security matters at arm’s length (Lanteigne and Hoogensen Gjørv 2020). More recently, however, global tensions have spilled over into Arctic affairs, for example, as the United States named China and Russia as threats to order and security in the Arctic (Office of the Undersecretary for Defense Policy 2019). Physical and biological changes in the Arctic Ocean are not the sole or even primary determinant in the ways the Arctic is entrained in global affairs, but environmental changes affect both reality and perception of an increasingly accessible and relevant Arctic, giving the Arctic increased prominence on the global stage and thus drawing greater interest, scrutiny, and concern.

## Discussion

The Arctic Ocean is changing. The implications of those changes are spreading far and wide. But not all are inevitable. Societies change their behaviors in response, and the combination of environmental and social change over time produces the types of outcomes we have described above. While predicting environmental changes is important, shaping the responses of human societies may be even more important.

First and foremost is whether global society will take heed of the changes occurring in the Arctic and decide to act in ways that reduce further change. In the long term, the largest uncertainties in climate models concern human choices (IPCC 2014). Today’s decisions will have effects for decades or longer, potentially limiting the choices that future generations have. Such behavior contravenes the letter and the spirit of sustainability (World Commission on Environment and Development 1987).

Second is whether those involved in decisions affecting the Arctic—those within the region and those elsewhere—will work towards a unified, coherent vision for the region, or continue to pursue disparate and at times conflicting paths (Spohr and Hamilton 2020). A single, universally supported vision is unlikely, but some broad principles could be adopted. How and under what circumstances are Arctic resources to be exploited? What conservation measures should be employed and how? At present, the human presence in the Arctic Ocean remains relatively modest, creating a false sense that society can both develop mineral and other resources and protect the environment, contrary to experience elsewhere in the world (Huntington 2021). Sustainability requires greater attention to tradeoffs among competing uses of marine resources and areas, locally and regionally as well as globally.

Third, governments of Arctic countries and others affecting the region face the choice of whether to respect the rights of Arctic Indigenous Peoples or whether to continue to pursue exogenous visions for the region. Commercial whale hunting earned large profits at the expense of depleted whale populations, starving of communities, and cultural loss (Bockstoce 1986). Colonization degraded self-determination and undermined cultural continuity (Stuhl 2016). In many ways, Arctic Indigenous Peoples are adjusting to change already (Johnson et al. 2016). While forcing Arctic Peoples to respond to continued environmental degradation is unethical (Loring 2013), unilateral actions to “protect” and “conserve” the Arctic contravene the spirit and letter of environmental justice (Mohai et al. 2009).

To help create an effective response to change, the findings of the Changing Arctic Ocean Programme need to be shared beyond the realm of oceanographers and climatologists and with broader society. Scientific findings need to be combined with other forms of knowledge, such as that of Indigenous Peoples, and incorporated into the policy discussions held by governments and influenced by the public at large. The alternative, in the Arctic as globally, is that scientists document what is changing but that information plays little or no part in how society responds. Those responses will not be simple or easy, but they will determine the outcomes of what is happening now in the Arctic Ocean. Those outcomes will affect everyone in the Arctic and around the globe.
